# Preparation of the RIPK3 Polyclonal Antibody and Its Application in Immunoassays of Nephropathogenic Infectious Bronchitis Virus-Infected Chickens

**DOI:** 10.3390/v14081747

**Published:** 2022-08-10

**Authors:** Guanming Tian, Yan Shi, Xianhong Cao, Wei Chen, Yueming Gu, Ning Li, Cheng Huang, Yu Zhuang, Guyue Li, Ping Liu, Guoliang Hu, Xiaona Gao, Xiaoquan Guo

**Affiliations:** 1 Jiangxi Provincial Key Laboratory for Animal Health, College of Animal Science and Technology, Jiangxi Agricultural University, Nanchang 330045, China; 2 School of Computer and Information Engineering, Jiangxi Agricultural University, Nanchang 330045, China

**Keywords:** RIPK3, NIBV, polyclonal antibody, immunoassays, Hy-Line brown chicks

## Abstract

Receptor interacting protein kinase 3 (RIPK3) is a vital serine/threonine kinase in regulating the programmed destruction of infected cells to defend against RNA viruses. Although the role of RIPK3 in viruses in mice is well characterized, it remains unclear where in nephropathogenic infectious bronchitis virus (NIBV) in chickens. Here, we use a self-prepared polyclonal antibody to clarify the abundance of RIPK3 in tissues and define the contributions of RIPK3 in tissue damage caused by NIBV infection in chickens. Western blot analyses showed that RIPK3 polyclonal antibody can specifically recognize RIPK3 in the vital tissues of Hy-Line brown chicks and RIPK3 protein is abundantly expressed in the liver and kidney. Moreover, NIBV significantly upregulated the expression levels of RIPK3 in the trachea and kidney of chicks in a time-dependent manner. In addition, the activation of necroptosis in response to NIBV infection was demonstrated by the coimmunoprecipitation (CoIP) experiments through RIPK3 in the necrosome, which phosphorylates its downstream mixed-spectrum kinase structural domain-like protein (MLKL). Our findings offered preliminary insights into the key role of RIPK3 protein in studying the underlying mechanism of organ failure caused by NIBV infection.

## 1. Introduction

Nephropathogenic infectious bronchitis virus (NIBV) is a single-stranded positive-sense RNA virus with strong renal tissue tropism in chickens and belongs to the gamma coronavirus family [1,2,3]. Initially, in the 1960s, IBV strains called nephrotic IBV that cause severe nephritis were first identified in the United States [4]. The typical signs NIBV-infected chicks show are swelling and pallor of the kidneys and urate deposition in the renal tubules, which are caused by impaired renal function, due to NIBV replication in the renal tubular epithelium [5,6]. It can induce high morbidity and mortality in chickens and further cause huge economic losses to the poultry industry worldwide, which is also the most important pathogen inducing visceral gout outbreak [7]. Most previous studies generally believe that inflammatory response, which is initiated by pathogen recognition receptors via the NF-κB signal pathway, plays a key role in tissue damage following NIBV infection [8,9]. However, in recent years, emerging evidence demonstrates that RIPK3-induced intense inflammatory cell death closely correlates with the development of virus diseases, as can be evidenced by coronavirus disease 2019 (COVID-19) [10,11,12]. Hence, studying the potential role of RIPK3 in impaired tissues following NIBV infection will contribute to a better understanding of the pathogenesis of NIBV.

RIPK3, a cytosolic serine/threonine kinase, is identified as the chief promoter of necroptosis because the necroptotic pathway can proceed via RIPK3 in the ablation of RIPK1 [13,14]. From within the necrosome, activated RIPK3 recruits the mixed-spectrum kinase structural domain-like protein (MLKL) and promotes its phosphorylation. In turn, the phosphorylated MLKL then translocates to the plasma membrane and leads to cell rupture [15,16]. Recently, accumulated studies reported that some viruses can directly or indirectly interact with RIPK3, thereby regulating the necroptosis pathway [17,18]. The viral protein encoded by the mouse cytomegalovirus (MCMV) M45 gene can directly inhibit virus-induced necroptosis by combining with RIPK3 through the RHIM structural domain, promoting its replication in host cells [19]. Similarly, influenza A virus (IAV) infection induces necroptotic pathway through a Z-DNA-binding protein 1 (ZBP1)/RIPK3-driven signaling axis [20,21]. These observations strongly bolster this view that RIPK3-dependent cell death functions as host defense to prevent viral infection. More importantly, RIPK3 also exhibits necroptosis-independent functions as a scaffold for interacting partners in apoptosis and pyroptosis [22]. These cell death pathways can perpetuate the release of cytokines and damage-associated molecular patterns, leading to intense inflammation [23,24]. Hence, unrestrained activation of RIPK3 will be detrimental to the host during virus infection [21,25]. However, the role of RIPK3 in NIBV infection is still not well understood.

At present, the antibody to RIPK3 from chickens has not been reported and there are no studies on the role of RIPK3 in tissue injury following NIBV infection. In the current study, the prepared polyclonal antibody RIPK3 was first applied to detect the expression levels of RIPK3 protein in different tissues of chicken. In addition, the changes in RIPK3 following NIBV infection were observed by the Western blot results. More importantly, we further explored whether necroptosis induced by NIBV occurs. All the studies indicated that the RIPK3 antibody produced in the present study is applicable to various immunodetections of chicken RIPK3 protein and it provides a basis for further study of the mechanism of kidney injury caused by NIBV infection.

## 2. Results

### 2.1. Construction of pET-32a-RIPK3 Prokaryotic Expression Vector

**Figure 1:**
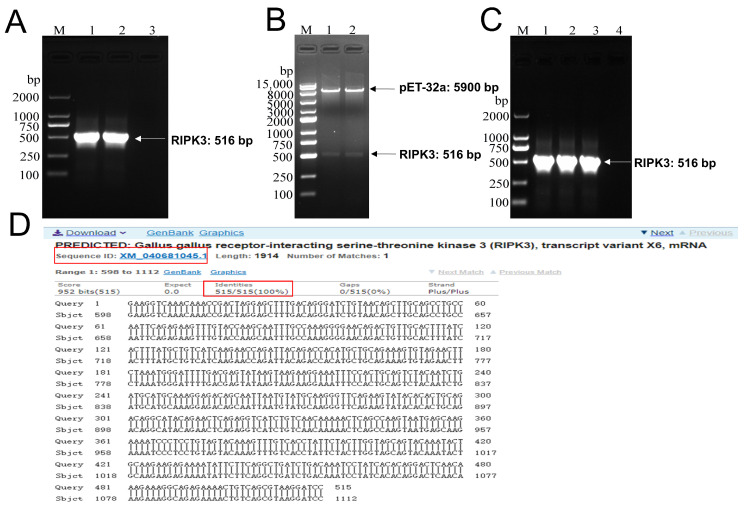
Construction of pET-32a-RIPK3 prokaryotic expression vector. (**A**) The coding sequence of the RIPK3 truncated protein gene was successfully obtained via PCR amplification; lane 1–2: RIPK3 gene (516 bp) was amplified at 56 °C; lane 3: negative control. (**B**) RIPK3 truncated protein gene was successfully inserted into the pET-32a (+) vector as confirmed by double-restriction digestion identification; lane 1–2: recombinant pET-32a-RIPK3 after double-enzyme digestion. (**C**) Colony PCR; lane 1–3: PCR products of RIPK3 truncated protein gene in bacterial liquid; lane 4: negative control. (**D**) Sequencing results of recombinant plasmids transferred into BL21 receptor cells.

According to the mRNA sequence of Gallus’ RIPK3 published online by GenBank (GenBank accession number: XM_040681043.1), its primers were designed through Primer 5 and the size of the PCR product was as large as the expected 516 bp (Figure 1A). The purified target gene was inserted into the double-enzyme-digested pET-32a plasmid and then transformed into the competent cell BL21. The positive samples identified by double-enzyme digestion and bacterial liquid PCR were sent to TsingKe Biotechnology Co (Beijing, China) for sequencing (Figure 1B,C). The results of the blast in Figure 1D show that the amplified sequence was highly consistent with the expected RIPK3 sequence.

### 2.2. Induced Expression, Identification, Purification, and Renaturation of RIPK3 Truncated Protein

**Figure 2:**
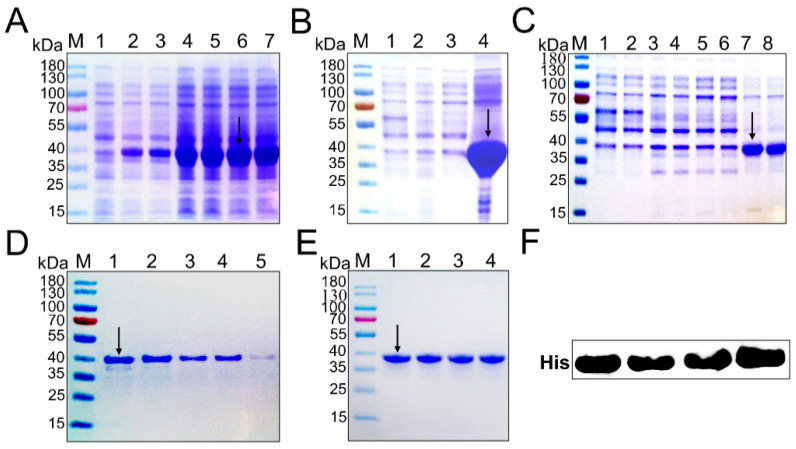
Expression and purification of RIPK3 protein. (**A**) Induction of recombinant protein expression at 37 °C; lane 1: total protein of non-induced pET-32a-RIPK3 protein; lane 2–7: total protein of pET-32a-RIPK3 protein was induced for 4 h, 6 h, and 8 h, respectively. (**B**) Identification of the expression form of recombinant protein induced at 37 °C after ultrasound; lane 1: total protein supernatant of non-induced pET-32a-RIPK3 protein; lane 2: total protein sediments of non-induced pET-32a-RIPK3 protein; lane 3: total protein supernatant of induced pET-32a-RIPK3 protein; lane 4: total protein sediments of induced pET-32a-RIPK3 protein. (**C**) Identification of the expression form of recombinant protein induced at 20 °C after ultrasound; lane 1–2: total protein supernatant of non-induced pET-32a-RIPK3 protein; lane 3–4: total protein sediments of non-induced pET-32a-RIPK3 protein; lane 5–6: total protein supernatant of induced pET-32a-RIPK3 protein; lane 7–8: total protein sediments of induced pET-32a-RIPK3 protein. (**D**) Determination of optimum imidazole concentration; lane 1–5: the target protein was eluted with imidazole eluting buffer (100, 200, 300, 400, 500 mM) at gradient concentration. (**E**) lane 1–4: the target protein was concentrated after dialysis; M: the standard protein Marker (15–180 kDa). (**F**) His tag of purified target protein was verified by Western blot. The short black arrows indicate RIPK3 protein bands.

In the LB/Amp liquid medium, recombinant RIPK3 protein was expressed in large quantities under the induction of 1.0 mM IPTG at 37 °C (Figure 2A). After sonication, we found that the expressed recombinant protein existed as inclusion bodies (Figure 2B). Then, we want to increase the soluble expression of the protein by inducing it at 20 °C to slow down the synthesis of the recombinant protein, but the target protein is still in the inclusion body (Figure 2C). The inclusion body protein dissolved in a solution containing 8M urea was purified using NI-NTA affinity column and then subjected to dialysis and renaturation (Figure 2D,E). Finally, the refolded His-RIPK3 fusion protein was incubated with primary antibody against His-tag and there was an obviously homogenous band corresponding to molecular weight (MW) of around 40 kDa (Figure 2F).

### 2.3. Antibody Titers Were Determined by Indirect ELISA

**Figure 3:**
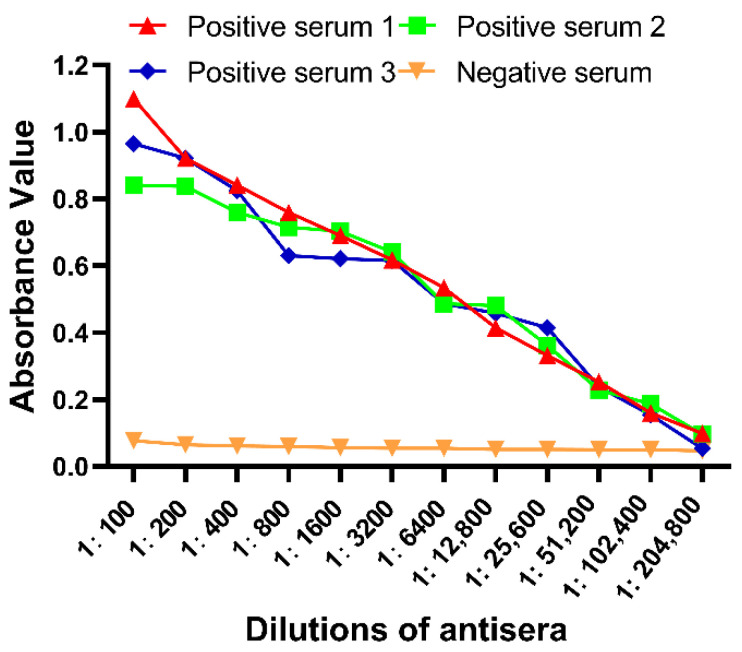
The absorbance of negative and positive serum with different dilutions was determined by ELISA. The ratio of the positive serum and the negative serum is greater than 2.1 and the titer of the antibody is the dilution factor of the antibody. X-axis: different dilution ratios of anti-RIPK3 serum using multiple dilution methods. Y-axis: the value of anti-RIPK3 serum at 450 nm wavelength. Red, green, and blue represent positive serum (serum obtained from rabbits after immunization) and orange represents negative serum (serum obtained from rabbits before immunization).

To assess the titer of rabbit anti-chicken RIPK3 protein antiserum, we used an enzyme-linked immunosorbent assay (ELISA) for RIPK3 antibodies. The purified RIPK3 truncated protein was immobilized on the ELISA plate at the optimal concentration (1.5 μg/mL) as an antigen. Further, when OD_positive_/OD_negative_ ≥ 2.1, the titer of the antibody is the dilution multiple of the antibody. The sera obtained 7 days after the third vaccination as positive serum showed a very strong specific antibody response in the rabbit, with a positive reaction at a dilution of 1:102,400. In contrast, the sera from the pre-immunization rabbit as negative serum showed only background-level antibody responses (Figure 3).

### 2.4. Histopathological Observation

**Figure 4:**
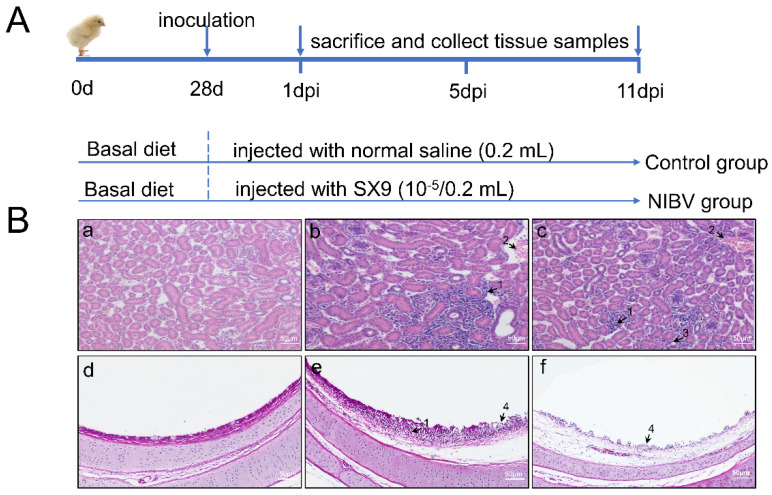
NIBV infection aggravates the histological lesions of tracheal tissues and renal tissues in the chicken. (**A**) The schematic illustrations of experimental design. (**B**) Histological analysis of chicken kidneys and tracheae. (**a**,**d**) is the normal renal tissue section and trachea tissue section, respectively; (**b**,**c**,**e**,**f**) are the representative images of tracheal tissues and renal tissues at 11 dpi showing histopathological changes in NIBV-infected chickens. 1: Serous fluid and inflammatory cell infiltration; 2: blood cell infiltration; 3: the absence of tubular structures; 4: most of the cilia shed and the mucosal epithelial cells were degenerated, necrotic, and shed.

Our experiments in histopathological observation revealed that NIBV infection aggravated the histological lesions of tracheal tissues and renal tissues in the chicken. As H&E staining showed (Figure 4B(a)), the renal cells in the control group were clear. The structure of renal tubules and glomerulus was obvious. Besides, tracheal surface epithelium with intact cilia and tightly packed muscle cells can be observed in the control group (Figure 4B(d)). In contrast, the NIBV-infected group has caused histopathological changes in renal tissues and tracheal tissues. There were prominent inflammatory cells and blood cells infiltrated in the interstitium outside the lumen under the treatment with NIBV infection. Further, the renal tubule structure was destroyed (Figure 4B(b,c)). In addition, shedding of ciliary and degeneration and necrosis of mucosal epithelial cells were observed in the trachea of chickens treated with NIBV (Figure 4B(e,f)).

### 2.5. Differential Expression of RIPK3 in Tissues

**Figure 5:**
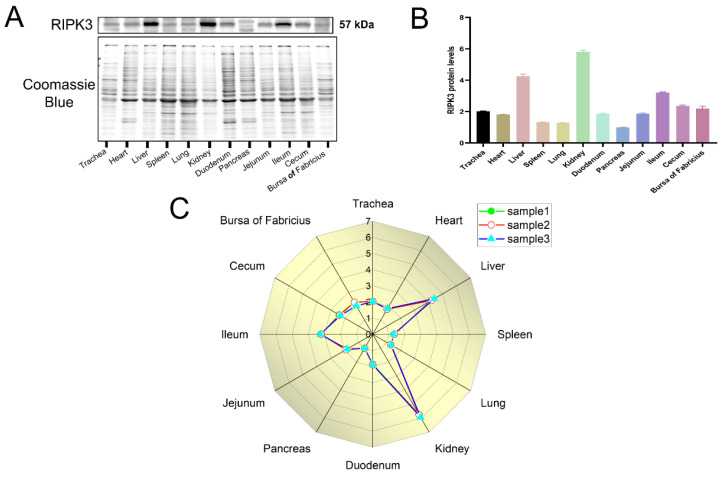
Expression levels of RIPK3 protein in different organs of 28-day-old Hy-line laying chickens (*n* = 3). (**A**) Western blot explores the expression levels of the target protein in twelve tissues in the chickens. (**B**) The expression levels of RIPK3 relative to the total protein stained with Coomassie Blue. (**C**) Radar chart analysis of RIPK3 protein levels.

To examine the dynamic expression of RIPK3 in various tissues in chickens, extracted total protein from tissues was performed in Western blot analysis. The samples represented 28-day-old Hy-Line brown laying hens. As shown in Figure 5, the protein levels of RIPK3 were highest in the kidney, followed by the liver and the ileum among the twelve tissues we measured (trachea, heart, liver, spleen, lung, kidney, duodenum, pancreas, jejunum, ileum, cecum, and bursa of Fabricius).

### 2.6. Effects of NIBV on RIPK3 Levels

**Figure 6:**
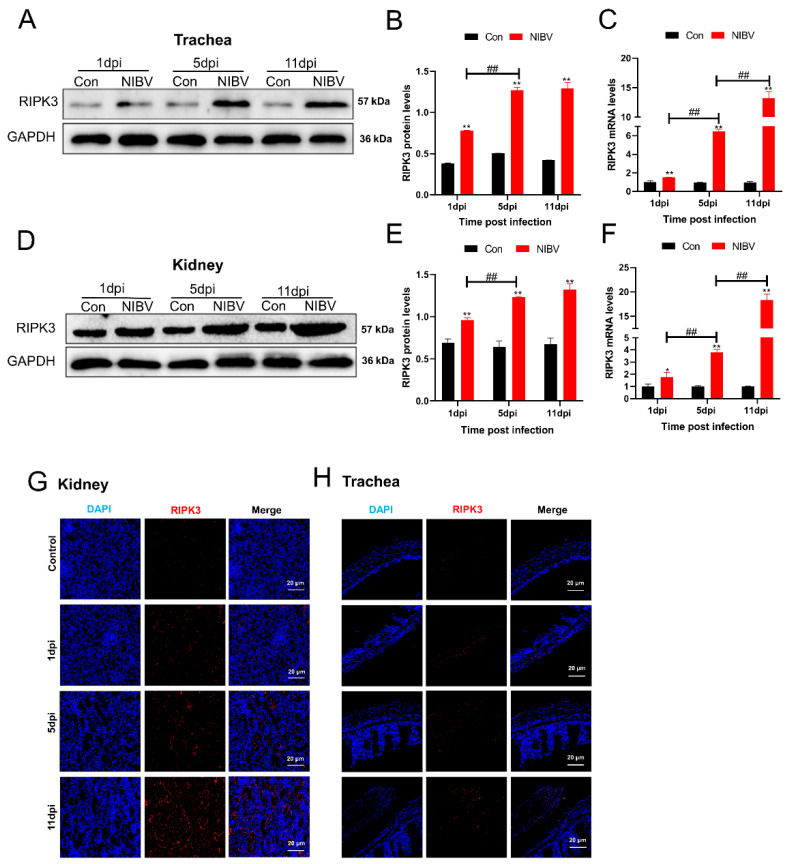
NIBV infection upregulates the protein expression levels of RIPK3 in the trachea and kidney. (**A**) The total protein extracted from tracheal tissues collected at 1-, 5-, and 11-days post infection was subjected to Western blot analyses. (**B**) Quantification of RIPK3 expression relative to gapdh. (**C**) RIPK3 mRNA levels of tracheal tissues were examined by real-time PCR. (**D**) After NIBV inoculation for 1 d, 5 d, and 11 d, the expression levels of RIPK3 in kidney tissues were detected by Western blot. (**E**) Quantification of RIPK3 expression in the kidney relative to gapdh. (**F**) The changes in RIPK3 mRNA levels in response to NIBV infection. (**G**,**H**) Fluorescence microscopy analyzed the fluorescence intensity of endogenous RIPK3 (red) in renal tissues (**G**) and tracheal tissues (**H**) after NIBV infection for 1 d, 5 d, and 11 d. Scale bar: 20 μm. The data in the results are expressed in the form of mean ± SD (*n* = 3). The data are expressed as mean ± SD. A difference was considered significant if *p <* 0.05. Statistical extreme significance was defined as *p <* 0.05 (*, #), *p <* 0.01(**, ##).

To further verify the specificity of the RIPK3 antibody and explore the effect of NIBV on RIPK3, the expressions of RIPK3 in the total protein of kidney and trachea homogenate were detected by Western blot analysis, real-time quantitative PCR, and immunofluorescence staining. As shown in Figure 6A,B,D,E, the protein levels of RIPK3 in NIBV-infected renal cells and tracheal cells exhibited an increase (*p <* 0.05) at 1, 5, and 11 days post infection. Similarly, we also observed that NIBV infection significantly upregulated (*p <* 0.05) the mRNA levels of RIPK3 compared with the control group (Figure 6C,F). Moreover, according to the results of immunofluorescence detection, we found that the fluorescence intensity of RIPK3 increased at 1 dpi, 5 dpi, and 11 dpi with time (Figure 6G,H).

### 2.7. NIBV Infection Induces Necroptosis

**Figure 7:**
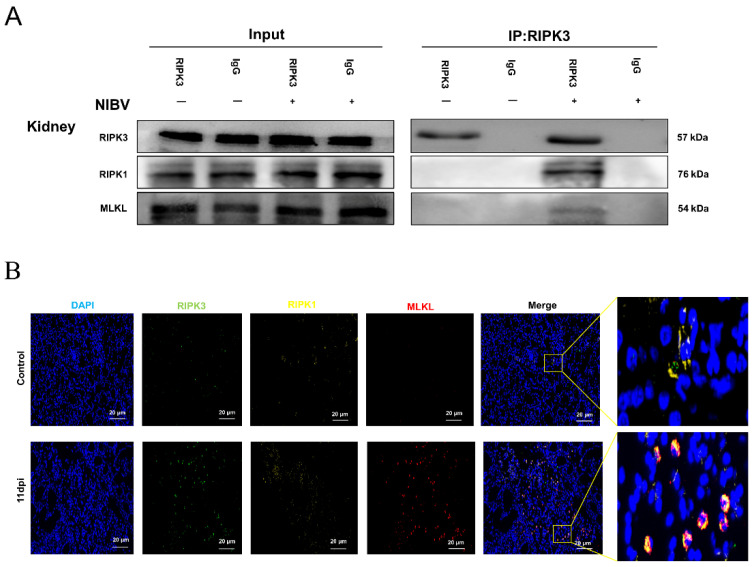
NIBV infection induces necroptosis. (**A**) Immunoprecipitation showed that RIPK3–RIPK1 and RIPK3–MLKL interactions were increased in the renal tissues after NIBV inoculation for 11 d. The whole-kidney tissue homogenate extracts were immunoprecipitated with prepared RIPK3 antibody as described in Materials and Methods 4.9. The immunocomplexes were analyzed by Western blot using the indicated antibodies. In addition, the Input group was subjected to SDS-PAGE followed by Western blot analysis of RIPK1, RIPK3, and MLKL. (**B**) Fluorescence microscopy analyzed the colocalization of the RIPK1 (yellow) and MLKL (red) with RIPK3 (green) in renal tissues after NIBV infection for 11 dpi. RIPK3 proteins were highly colocalized with RIPK1 and MLKL compared with the control group. Scale bar: 20 μm.

Given that the RIPK1–RIPK3–MLKL interactions are reported to be involved in the induction of necroptosis [26,27], we performed the CoIP experiments to investigate whether necroptotic response occurs during NIBV infection in the kidneys of chickens. As shown in Figure 7A, the Input group and IP group represented 10% of kidney homogenate extracts used in the immunoprecipitation step and immunoprecipitation with added RIPK3 antibody, respectively. Western blotting showed that endogenous RIPK3 co-immunoprecipitated with RIPK1 and MLKL after NIBV infection (Figure 7A, compare lane RIPK3—with RIPK3 + in the IP group). Coincidently, subsequent immunofluorescence colocalization analysis assessing the percentage of overlapping area in the NIBV group revealed that the RIPK3 proteins were highly colocalized with RIPK1 and MLKL compared with the control group (Figure 7B). These results suggest that NIBV infection induces necroptosis in the kidney of chickens.

## 3. Discussion

The pET vectors are considered to be one of the most powerful vector systems for cloning and expressing exogenous genes in *E. coli* (*Escherichia. coli*) [28]. Target genes inserted in pET plasmids are under the control of the T7 promoter and expression is induced by providing T7 RNA polymerase in the host cell. T7 RNA polymerase has a high degree of selectivity and activity, which helps to locate almost all cellular resources on the expression of specific genes [29]. Our research found that the pET-32a (+) vector tended to facilitate the efficient expression of RIPK3 protein relative to other vectors. The reason for this is that the pET-32a (+) vector is designed to express protein sequences fused with His-tag, facilitating purification and identification of the target protein. Furthermore, the core of prokaryotic expression depends not only on the vector but also on the host. *E. coli* host is the most preferred host in the expression of the exogenous gene, due to its characteristics of rapid reproduction, relative cheapness, and high efficiency [30]. In this study, the truncated recombinant RIPK3 protein was expressed by transforming the constructed pET-32a-RIPK3 plasmid into BL21 (DE3)-competent cells and induction with IPTG. BL21 (DE3), as a member of the *E. coli* host, has long had an important role in recombinant protein production. This is because the bacterial strain has several prominent features, including rapid cell growth in minimal media, low protease abundance, and suitability for high-density culture [31]. The RIPK3 antibodies now commercially available in the market are generally based on human protein sequences as recombinant proteins or designer peptides as antigens, which leads to the possibility of not recognizing the RIPK3 protein in chickens because of the large difference in RIPK3 protein homology between humans and chickens. In this article, we revealed that the low-cost RIPK3 antibody can be used to detect RIPK3 protein in chickens. We also observed that RIPK3 protein expression varied among tissues, with the highest expression in the kidney.

Nowadays, NIBV infection is one of the common causes of gout in the poultry industry, resulting in huge economic losses [8,32]. Upon infection with NIBV, the respiratory system and urinary system in chickens is damaged and key reference indicators (uric acid, creatinine) of renal impairment peaked at 11 days [33,34]. This study aimed to investigate what role RIPK3 plays in the effect of NIBV on the trachea and kidney tissue of chickens. Our results show that the expression levels of RIPK3 in post-infection tracheal and renal tissues gradually rose with the increasing duration of infection and NIBV activates RIPK3-driven necroptosis.

RIPK3 has established roles in either host defense or viral pathogenesis in programmed cell death pathways. On the one hand, infected cells are prevented from becoming viral replication niche and provide viral antigens and damage molecular patterns to stimulate adaptive immune responses against viral infection, while on the other hand, RIPK3-driven programmed cell death contributes to disease development during viral infection, leading to inflammatory responses and tissue damage [17,35]. Upon virus infection, viral PAMPs are detected by host pattern recognition receptors (PRRs) [36]. The activation of PRRs can often initiate RIPK3 and the pathways engaged by RIPK3 that drive cell death [17,35]. Inflammatory products consisting of DAMPs, alarmins, and inflammatory cytokines are released from lysed cells and can perpetuate cytokine storm and organ damage [35]. The process of coronavirus infection leads to a massive release of inflammatory cytokines forming a cytokine storm as well as acute lung injury induced by intense inflammatory cell death and RIPK3 is involved in this process [25,37]. Similar results were observed in kidney tissues after NIBV infection: significant elevation of pro-inflammatory cytokines, such as TNFα, IL-6, IL-18, IFN-γ, and renal injury [34,38]. Consequently, based on these observations, it would be interesting to investigate the role of RIPK3 in NIBV infection. In our study, we used successfully prepared RIPK3 polyclonal antibodies to perform Western blot analyses and immunofluorescence detection found that NIBV significantly upregulated RIPK3 protein levels. Based on experimental data from cell culture and mouse models, the activation of necroptosis is consistent with the increased expression of RIPK3 [39]. Therefore, we suspect that NIBV may activate RIPK3-dependent activation of necroptosis in the renal tissues, which was proved by the Co-IP and immunofluorescence colocalization [40].

## 4. Materials and Methods

### 4.1. Experimental Model and Subject Details

Three hundred Hy-Line brown chicks, 27 days old, were randomly allocated into two groups: the control group and the NIBV infection group, which were fed in two feeding rooms, respectively. To avoid cross-infection, breeders were also split into two groups. At 28 days of age, each chick in the NIBV infection group received SX9 strain (10^−5^/0.2 mL) allantoic fluid via the ocular and nasal route, while the control group received the same dose of normal saline. The inoculum was administered as drops, using a sterile syringe. The strain of NIBV (SX9) was isolated and stored at the College of Animal Science and Technology Jiangxi Agricultural University. Reverse transcription polymerase chain reaction (RT-PCR) and bacterial culture showed that there were no other pathogens. The specific sequence of the laboratory-isolated strain can be seen on NCBI (Accession number: MN707951.1). The virus titers were determined as 50% embryo lethal doses (ELD50). Trachea samples and kidney samples were collected from 8 chickens in each group at 1-, 5-, and 11-days post-infection (dpi). In the animal laboratory at the College of Animal Science and Technology of Jiangxi Agricultural University, all chicks were raised by the guidelines of laboratory feeding standards.

### 4.2. Construction of pET-32a-RIPK3 Expression Vector

RNA was harvested with TRIzol reagent (TransGen Biotech, Beijing, China) from Hy-Line brown chicks according to the manufacturer’s instructions and reverse transcription was executed with Reverse Transcriptase Kit (TransGen Biotech, Beijing, China). PCR was then performed to amplify the RIPK3 protein gene using the following primers: (Fwd) 5′-CCGGAATTC GAAGTAGATATTTGGAGCAG-3′, (Rev) 5′-CCCAAGCTT TGATGAGGTAAGGGATGT-3′ (The italics represent EcoRI, HindIII restriction sites). The pET-32a (+) (Takara Bio Inc, Shiga, Japan) plasmid and amplified PCR products were subsequently digested by double endonuclease (EcoRI, HindIII: Takara Bio Inc, Shiga, Japan) followed by purification and then ligated together by T4-DNA ligase (Takara Bio Inc, Shiga, Japan) at 4 °C overnight. Subsequently, BL21-competent cells (TianGen Biotech, Beijing, China) following the transformation of the recombinant plasmid were cultured on LB/Amp solid plate. A single positive colony from the plate was inoculated into the liquid medium. Finally, PCR-positive samples were sent for sequencing.

### 4.3. Induction and Purification of pET-32a-RIPK3 Truncated Protein

After sequencing to confirm that it is correct, the expression of recombinant RIPK3 truncated protein was induced at 37 °C and 20 °C, respectively, adding 1 mM isopropyl 1-thio-beta-d-galactopyranoside (IPTG) into liquid LB/Amp medium. Expression levels of the induced RIPK3 were identified with 12% SDS-PAGE and purified with Ni-NTA 6FF Sefinose Resin Kit (Sangon Biotech, Shanghai, China). Subsequently, Western blot analysis with anti-His tag further verified that the protein purified is the target protein containing His-tag. Finally, the target protein eluted by the optimal concentration of imidazole was subjected to dialysis and renature.

### 4.4. Preparation of RIPK3 Truncated Protein Polyclonal Antibody

First, 5 mL of blood was collected from each rabbit to separate the serum, which was used as the negative control for subsequent antibody detection. RIPK3 truncated protein and the same amount of complete Freund’s adjuvant (Sigma-Aldrich Biological Chemistry, St. Louis, MO, USA) were used as antigens after being completely emulsified and rabbits were immunized by intradermal injection. Second and third emulsification was performed with equal amounts of Freund’s incomplete adjuvant and RIPK3 truncated protein, followed by immunization every 10 days. After three injections, blood was obtained from the heart and centrifuged at 4000 rpm for 5 min at 4 °C to separate the antiserum. The titer of purified antibodies was measured using an enzyme-linked immunosorbent assay (ELISA).

### 4.5. Histopathology

Kidneys and tracheae were fixed with 10% formalin, then embedded in paraffin. Upon mounting and deparaffining, the sections stained with hematoxylin and eosin (H&E) were observed under a microscope.

### 4.6. Western Blotting

The total protein extracted from the collected chicks’ tissue samples was subjected to Western blot analysis to evaluate the specificity of the polyclonal antibody RIPK3. Western blot experiments were performed as in the previous study [41]. Briefly, proteins from tissues were separated by electrophoresis through 12% SDS-PAGE. Following the transfer of proteins onto the PVDF membrane (Sigma-Aldrich Biological Chemistry, St. Louis, MO, USA), the blotted membrane was then sealed in the blocking buffer (Sorfa Life Science, Zhejiang, China) for 20 min and incubated with prepared antibody: RIPK3 (1:500). Notably, as the content of housekeeping protein GAPDH in various tissues is different, total protein levels stained with Coomassie in place of GAPDH were used as the loading control when Western blot detection of RIPK3 protein in tissues was performed [42,43].

### 4.7. Quantitative PCR Analysis

Total RNA was isolated using TRIzol reagent (TransGen Biotech, Beijing, China), following the manufacturer’s protocols. Reverse transcription was executed using the First-Strand cDNA Synthesis Kit (TransGen Biotech, Beijing, China). Real-time PCR was performed using the Quant StudioTM 7 Flex Real-time Quantitative PCR detecting system (Thermo Fisher Scientific, Waltham, MA, USA). GAPDH was chosen as the internal control for the calculation of the relative abundance of RIPK3 mRNA with the 2^−ΔΔCt^ method.

### 4.8. Immunofluorescence Assay

Paraffin sections of chicken tissues were routinely dewaxed, dehydrated in gradient alcohol, and subjected to antigen repair. Subsequently, the sections were incubated with 10% bovine serum protein for 30 min and the indicated primary antibodies were added dropwise and incubated overnight at 4 °C. After washing in PBS, the slices were incubated with FITC-coupled goat anti-rabbit IgG (Servicebio, Wuhan, China, 1:1000) at room temperature for 40 min, followed by staining with DAPI (4′,6-diamino-2-phenylindole, Servicebio, Wuhan, China). Finally, a fluorescent microscope was used to capture the images.

### 4.9. Coimmunoprecipitation

For immunoprecipitation, tissue samples were lysed in the IP lysis buffer supplemented with protease and phosphatase inhibitor (Solaibao Technology Co., Beijing, China). Tissue lysates following incubation on ice for 30 min were to obtain supernatant via centrifugation at 12,000 rpm for 5 min at 4 °C. To remove nonspecific heteroproteins and reduce the background, protein A/G agarose (Yeasen Technology Co., Shanghai, China) was added to the extracted proteins. Subsequently, the centrifuged supernatant was transferred to a separate 1.5 mL Eppendorf tube and incubated with antibody against RIPK3 and protein A/G agarose overnight at 4 °C followed by removing nonspecific binding with wash buffer. Finally, the precipitation after centrifugation was immunoblotted with the primary antibodies: RIPK1 and MLKL. RIPK1 was purchased from Wanlei biotechnology (Shenyang, China). MLKL was obtained from Santa Cruz Biotechnology (Santa Cruz, CA, USA).

### 4.10. Statistical Analysis

Regular statistical analysis was performed with GraphPad Prism 8 software (GraphPad Software, La Jolla, CA, USA). One-way analysis of variance (ANOVA) and t-test were used to compare the results. The data are presented as the mean ± standard deviation (SD). A difference was considered significant if *p* < 0.05. Statistical significance was defined as *p <* 0.05 (*, #), *p <* 0.01(**, ##).

## 5. Conclusions

In conclusion, the production of a polyclonal antibody against chicken RIPK3 with high specificity was described via a simple and cost-effective method and this antibody can be used in Western blot, immunofluorescence staining, and Co-IP experiments. Our results showed that the content of RIPK3 protein varies in different tissues of chickens. Moreover, NIBV significantly upregulates RIPK3 protein levels and RIPK3-mediated necroptosis following infection with NIBV occurring in the kidney. Collectively, these findings provide novel insight for understanding the mechanism of tissue damage caused by NIBV.

## Data Availability

Not applicable.
